# Reasonable Expectations of Privacy and Disclosure of Health Data

**DOI:** 10.1093/medlaw/fwz009

**Published:** 2019-04-25

**Authors:** Mark J Taylor, James Wilson

**Affiliations:** 1 Associate Professor in Health Law and Regulation, Melbourne Law School, University of Melbourne, Australia; 2 Senior Lecturer, Department of Philosophy, UCL, London, UK

**Keywords:** Duty of confidentiality, reasonable expectations, consent, confidential patient information, health data, privacy

## Abstract

The law of confidence allows for a range of defined circumstances in which confidential patient information (CPI) can be disclosed without breach of confidence—including statutory gateway and overriding public interest. Outside such circumstances, current guidance to health professionals (the ‘standard account’) assumes that CPI can only be lawfully disclosed with patient consent. This article argues that the standard account has not yet caught up with judgments, post the Human Rights Act 1998 coming into force, which have reinterpreted the law of confidence in the light of Article 8 of the European Convention on Human Rights. In particular, the article explains the significance of the concept of a ‘reasonable expectation of privacy’ to an action for breach of confidence and thus to legal liability for disclosure of health data. It argues that conformity with a reasonable expectation of privacy provides an alternative account for the lawful disclosure of CPI, and may provide a more sustainable and authentic approach to meeting obligations under the law of confidence than the standard account. The article concludes with recommendations for an evolution of the standard account in a way that could allow restatement of associated concepts (such as consent) free from particular pressure to bend them out of shape. The evolution proposed continues to bring to the fore the patient perspective and allows protection of their ‘reasonable expectations’ regarding uses of data collected about them rather than those of the profession.

## I. INTRODUCTION

It has been consistently stated in guidance to health professionals (in what we shall call the ‘standard account’) that any flow of identifiable health information absent legal requirement, statutory gateway, or overriding public interest, needs to be on the basis of an individual’s explicit or implied consent. In this article we describe and explain the significance of the concept of a ‘reasonable expectation of privacy’ to the equitable action for breach of confidence and thus to legal liability for disclosure of health data. We argue that conformity with a reasonable expectation of privacy provides an alternative account for the lawful disclosure of identifiable health information. We explain why it may provide a more sustainable and authentic approach to meeting obligations under the law of confidence than the standard account. Our primary focus is disclosure for purposes associated with an individual’s direct care, though in so doing we also clarify the duty of confidence beyond such purposes.

Our argument for a radical review of the standard account is motivated not only by recent revelation of its limitations but also by recognition that it has not tracked legal developments. We take our cue from Dame Fiona Caldicott, National Data Guardian for Health and Social Care, and her call for a patient’s reasonable expectations, regarding confidential data flows, to be respected. Through consideration of case law, including the case of *R (W, X, Y and Z) v Secretary of State for Health and Secretary of State for the Home Department*,^1^ we chart an alternative to the standard account that foregrounds the legal concept of a reasonable expectation of privacy. Clarity on the appropriate legal tests for lawful disclosure is crucial at this time. There is significant innovation in the healthcare context and uncertainty regarding when the standard account permits data to flow in cases of novel arrangements. The immediate risk is for the standard account to either stultify change or for its interpretation to be strained in ways that diminish protection. One advantage of focussing upon the law of confidence and data protection legislation is that both have application across the UK and unite otherwise relatively distinct governance arrangements.

Our aim in foregrounding the test of ‘reasonable expectations’ is not to undermine the significance of the routes to lawful use, such as consent, recognised by the standard account. On the contrary. We will argue that our account allows for their authentic restatement within a modern context. For example, it allows for consent to be claimed only where it is real.^2^ Our account also, importantly, identifies a substantive restraint (protecting patients from unreasonable uses of data and upholding the principle of ‘no surprises’) where overextension of the concept of implied consent would provide none. We argue that this not only better reflects the approach taken by the courts when considering the duty of confidence but, with appropriately robust and compelling leadership, may result in better protection for the values of respect for autonomy and human dignity underlying privacy protection in a modern healthcare system.

The article consists of four parts. In part I, we consider the standard account and some of the limitations revealed in practice, and we explain why it sits uneasily with a real consent to data flows necessary to deliver care in the context of a modern healthcare system. With tightening requirements for valid consent in other areas of law, in particular data protection law, consent requirements associated with the standard account are increasingly discordant. In part II, we describe the significance of the concept of a reasonable expectation of privacy to an action for breach of confidence. We consider how a reasonable expectation is established and clarify components eg whose perspective counts. In part III, we further explore the internal and external limits on scope and content of the concept of reasonable expectation by considering its relationship to jurisprudence concerning Article 8(1) of the European Convention on Human Rights (ECHR) and the right to respect for private and family life. In part IV, we consider the implications of foregrounding the concept of reasonable expectation of privacy in professional guidance. At this point we briefly pivot, turning from constructing a positive argument for change, to acknowledge and rebut an alternative evolution to the one we propose. Specifically, we address the suggestion that what really matters in law is not compliance with the standard account but whether a professional’s conscience would be troubled by the disclosure in question (the ‘conscience test’). While the ‘conscience test’ neither best reflects the current judicial approach, nor adequately demonstrates respect for the underlying values, the emergence of this as an alternative proposition provides further evidence of dissatisfaction with the standard account and the urgent need for leadership. We conclude with recommendations for an evolution of the standard account to recognise the central significance of a reasonable expectation of privacy, in a way that could allow restatement of associated concepts (such as consent) free from particular pressure to bend them out of shape, but without a return to historic tests of unconscionability. The evolution we propose continues to bring to the fore the perspective of the patient or service user and allows protection of their ‘reasonable expectations’ regarding uses of data collected about them rather than those of the profession.

## II. PART I—THE STANDARD ACCOUNT

The National Health Service (NHS) Code of Practice on Confidentiality is being revised. The most recent published version dates back to 2003 and contains what we will describe as ‘the standard account’. Two paragraphs of the Code in particular may be taken to summarise relevant elements:

Where patients have been informed of:

a. the use and disclosure of their information associated with their healthcare; and

b. the choices they have and the implications of choosing to limit how their information may be used or shared;

then explicit consent is not usually required for information disclosures needed to provide that healthcare. Even so, opportunities to check that patients understand what may happen and are content should be taken.^3^

*Where the purpose is not directly concerned with the healthcare of a patient however, it would be wrong to assume consent*. Additional efforts to gain consent are required or alternative approaches that do not rely on identifiable information will need to be developed. ^4^ (emphasis added)

The standard account persists through to the 2017 revision to the most recent General Medical Council (GMC) guidance on confidentiality. This advises doctors:

You may disclose personal information without breaching duties of confidence when any of the following circumstances applies.

a. The patient consents, whether implicitly for the sake of their own care or for local clinical audit, or explicitly for other purposes

[…]

d. The disclosure is required by law, or the disclosure is permitted or has been approved under a statutory process that sets aside the common law duty of confidentiality

e. The disclosure can be justified in the public interest^5^

The GMC guidance continues:

When disclosing information about a patient you must:

a. …

b. Be satisfied that the patient:

i. Has ready access to information explaining how their information will be used for their direct care or local clinical audit, and they have the right to object

ii. Has not objected

c. Get the patient’s explicit consent if identifiable information is to be disclosed for purposes other than their direct care or local clinical audit, unless the disclosure is required by law or can be justified in the public interest.^6^

There are three things to note about the consent required by the standard account. First, only the use of confidential information for a purpose *beyond* an individual’s ‘direct care or local clinical audit’ requires an *explicit* consent. ‘Explicit consent’ is generally taken to require a positive statement, either written or oral, that unambiguously authorises something to occur that otherwise might not have been permitted.^7^ Where the use is for individual direct care (or local clinical audit) purposes, then consent may be implied. Where consent is implied, the consenting person’s authorisation is signalled through conduct. In the case of consent to treatment, the classic example would be rolling up one’s sleeve in order to receive an injection.^8^ In the case of consent for the sharing of confidential information, a common case would be where a patient has a consultation with a GP, and is referred to a specialist, where the patient’s act of agreeing to the referral is taken to imply consent for relevant portions of the patient’s confidential information to be shared with the specialist. It is important to distinguish implied consent, which is real but signalled through conduct, from hypothetical or presumed consent—where a person has not really consented, but it is thought that it is legitimate to act as if they have (perhaps because it is thought that they would consent if asked in appropriate circumstances).^9^ The implication of the 2003 Guidance is that there may be circumstances in which it is *not* wrong to *assume* consent to be implied through receipt of care, when data are used only for individual care purposes.^10^

Secondly, although the information requirements may be the same for an explicit or an implied consent in theory, the practice is quite different. The information requirements of a real consent under common law have been considered more extensively by one of us elsewhere.^11^ For present purposes it is sufficient to note that, although the term ‘explicit’ or ‘implied’ refers only to the nature of the signal (and any consent requires a person know—at least in broad terms—what they are agreeing to when he or she signals agreement^12^) the act of obtaining an explicit consent foregrounds communication in ways that reliance on an implied consent does not. The 2017 Guidance indicates that, in the context of individual direct care, one need only ensure the patient ‘has ready access to information explaining how their information will be used for their direct care or local clinical audit, and they have the right to object’. This is perhaps a reflection of the impracticability, recognised in 2017, of routinely checking that patients have ‘read and understood’^13^ leaflets on confidentiality and information disclosure for care. In any case, it is doubtful that patients are fully aware of all the uses of data that currently take place on the basis of an ‘implied consent’.^14^ This undermines any claim that such consent is real.

Thirdly, the standard account presumes a clear distinction between disclosures of confidential information for purposes directly related to an individual’s care and disclosures for other purposes. It is perhaps this assumption that has been most rigorously challenged by new models of care. With healthcare organisations committed to the principle of ‘collect once, use many times’^15^ and with increasingly complex data-flows in a modern NHS, implied consent is widely thought to have become overstretched and ambiguous in its boundaries. In 2013, the *Caldicott 2 Review* (Review) noted that ‘there was limited awareness of the boundaries of implied consent both among health and social care professionals who rely on it and other staff who feel it may apply to their practices’.^16^ The Review aimed to provide some clarity by making a distinction between direct and indirect care,^17^ and establishing a claim about practice that has been broadly followed, namely that confidential health information about an individual may be shared on the basis of implied consent only with other health and social care professionals who have a legitimate relationship with the patient, and only in support of that individual’s direct care.^18^ A significant plank of the argument in favour of the legitimacy of such sharing for the purposes of individual direct care was that doing so accorded with patients’ reasonable expectations about how information about them would flow around a healthcare system.^19^

One unintended consequence of this move to clarification was to foster the impression that if an activity could be described as direct care, then that activity would by definition be one for which implied consent could be inferred. The net result appears to have been a significant expansion in the way that some institutions define direct care. In one prominent case, Royal Free London NHS Foundation Trust shared the records of 1.6 million NHS patients with DeepMind for the development and testing of an App for detecting acute kidney injury, it argued, on the basis of implied consent for direct care.^20^ This was despite the fact that at the time that the data was shared, no steps were taken to make patients aware of this fact, and that only a small percentage of the 1.6 million patients would ever develop an acute kidney injury.^21^ This led the National Data Guardian (NDG) to explain that:

when work is taking place to develop new technology this cannot be regarded as direct care, even if the intended end result when the technology is deployed is to provide direct care. Implied consent is only an appropriate legal basis for the disclosure of identifiable data for the purposes of direct care if it aligns with people’s reasonable expectations, *i.e.* in a legitimate relationship.^22^

New models of care are placing further strain on the idea of implied consent in other ways. In some cases it is reportedly inhibiting data sharing that may improve quality of care but does not readily conform to the standard account. For example, within genomic medicine it is argued that best practices in care provision will require much wider sharing of identifiable information than would seem to be justified by the ‘legitimate relationship’ test that the NDG had envisaged.^23^

In short, it is not clear that implied consent could always provide a sustainable way forward for sharing confidential health information for the purposes of patient care as new models of care continue to emerge. Consent can provide a principled way of respecting individual autonomy only where the choices accorded to individuals are real; it is disrespectful to individuals’ autonomy to claim to be acting on the basis of their consent when the conditions required for real consent are not in place. Rather than stretch a valid conception of implied concept out of shape, the standard account could evolve to recognise the role of ‘reasonable expectation’ as an alternative legal basis for processing in some circumstances. Consent may not always be necessary *if* the use and disclosure of information can be shown to be consistent with a ‘reasonable expectation of privacy’—consistent with respect for underlying values of individual autonomy and human dignity. If persons would not be surprised to learn that information had been used for a particular purpose, even if they did not consider themselves to have positively signalled consent to that use, then we may protect social licence in processing for diverse purposes without overburdening the consent process.^24^

## III. PART II—REASONABLE EXPECTATION OF PRIVACY AND THE DUTY OF CONFIDENCE

### A. What is the Significance of a ‘Reasonable Expectation of Privacy’ in this Context?

Lord Nicholls in *Campbell* v *MGN* (2004) noted that:

The common law or, more precisely, courts of equity have long afforded protection to the wrongful use of private information by means of the cause of action which became known as breach of confidence. A breach of confidence was restrained as a form of unconscionable conduct, akin to a breach of trust. Today this nomenclature is misleading. … Now the law imposes a ‘duty of confidence’ whenever a person receives information he knows or ought to know is fairly and reasonably to be regarded as confidential.^25^

When determining when information is protected from wrongful use, Sir Anthony Clarke MR, in the case of *Murray v Express Newspapers plc*, summarised the relevant legal principle thus: ‘Essentially the touchstone of private life is whether in respect of the disclosed facts the person in question had a reasonable expectation of privacy’.^26^

If disclosure of personal information interferes with a reasonable expectation of privacy, without legal justification, then a legal wrong will be committed. Application of this apparently simple test raises a number of questions. Amongst them is whether the test is now the *only* way to establish a duty of confidence under English Law. In *Re JR38*, Lord Kerr preferred a more nuanced approach to one that held the test of reasonable expectation to be the ‘be all and end all’.^27^ However, despite cautioning against blinkering oneself to other relevant considerations, Lord Kerr did not deny that when there *was* a reasonable expectation of privacy, then relevant legal duties would ordinarily be engaged.^28^ Indeed, the test of ‘reasonable expectation’ now seems to be widely regarded as foundational to any assessment of a claim of breach of confidence or misuse of personal information.^29^ As Lord Clarke, also in *Re JR38*, said:

All the domestic cases support the proposition that, as Lord Nicholls put it, the touchstone of private life is whether the person in question had a reasonable expectation of privacy or, as Lord Sumption JSC put it in *Catt*, the test for what constitutes private life is whether there was a reasonable expectation in the relevant aspect.^30^

If a reasonable expectation of privacy is now foundational to a breach of confidence claim, then questions arise as to how the scope and content of the expectation is to be determined? We note at the outset Lord Kerr’s admonishment that a proper construction of relevant case law does not:

partake of an approach which starts the inquiry … in a context-free way, whether there was a reasonable expectation of privacy. Rather, it commends an examination of all the circumstances of the case in order to determine whether such an expectation can be said to exist.^31^

If we are not to arbitrarily determine what constitutes a ‘reasonable expectation’ in a ‘context-free’ way, then how should we approach examination of ‘all the circumstances of the case’?

### B. How is a Reasonable Expectation of Privacy Determined?

When applying the common law, it is trite to note that the courts seek consistency through reference to precedent. Here, inquiry is hampered by the fact that relatively few cases have considered the detailed contours of a reasonable expectation of privacy when data is disclosed for the purposes of individual direct care. For example, many seminal cases^32^ have involved identifiable information being disclosed to the world at large. Sharing with someone who continues to owe a duty of confidence (such as a health professional) will likely be treated differently from sharing with the world at large.^33^ To identify circumstances material to the breadth of the duty owed in the healthcare context, we must look to existing case law and draw inferences where we can.

We might approach such examination in two fundamentally different ways: they might be summarised as attempts to describe the boundaries of a reasonable expectation of privacy from the inside out or the outside in. That is to say, one could attempt to identify circumstances relevant to (self-limiting) the breadth of the duty (an internal approach), or identify other external considerations relevant to limiting the duty (an external approach), eg existence of an overriding public interest in the circumstances.^34^ In fact, as we will see, in order to complete our picture, we will have to do both. We start with the internal approach and describe considerations relevant to whether the concept of reasonable expectation of privacy is, or is not, engaged. Even taking this approach, it is clear that it is possible for the test of reasonable expectation of privacy to be failed: there will be circumstances in which there is no reasonable expectation of privacy. We unpack the critical viewpoint privileged by law in the following way.

First, we will consider the *perspective* that the courts consider relevant: from whose perspective is the reasonableness of an expectation of privacy to be assessed? A restricted answer will determine the breadth of any obligation owed. Second, we begin to consider the features of *circumstance* considered material from that perspective. We note that some of these features, such as non-triviality, may also operate as self-limiting (internal) constraints on the extent of any duty owed.^35^ In addition, we recognise the significance of the underlying values considered to be at stake by the courts. In this respect, there has been some development in the law over the past 20 years.

In part III of the article, we continue this consideration by widening our lens. Here we look at the impact of the ECHR and the Human Rights Act 1998 on the structure of relevant legal argument and, importantly, on the values that the law seeks to protect. The structure of argument set up by the ECHR underlines the importance of the distinction between an internal and external limit on the breadth of the duty of confidence. The distinction is important because whether the duty of confidence is limited by the internal reach of a reasonable expectation, or external factors outweighing any prima facie duty in the circumstances, determines the requirements to be met by any seeking to loosen the restraining effect of the law.

#### 1. Perspective

The courts could approach the question of reasonable expectation from at least three different perspectives. First, they could consider it from the perspective of the person receiving the information. This is the perspective implied by Lord Nicholls in *Campbell* v *Mirror Group Newspapers Ltd*: ‘Now the law imposes a “duty of confidence” whenever a person receives information he knows or ought to know is fairly and reasonably to be regarded as confidential’.^36^

Secondly, the perspective could be that of an objective third party learning of the disclosure, eg a reader of a revealing newspaper story. Lord Hope, again in *Campbell*, suggested this to have been the perspective adopted by the Court of Appeal in the same case. He was critical. In his opinion, ‘[t]he mind that has to be examined is that, not of the reader in general, but of the person who is affected by the publicity’.^37^

This then takes us to the third alternative: the courts could consider the perspective of a reasonable person who was in the position of the person whose information is being revealed. This is clearly Lord Hope’s view. The relevant question is ‘what a reasonable person of ordinary sensibilities would feel if she was placed in the same position as the claimant’.^38^

It is important to underline that this is not a subjective view. It is not determinative whether a particular individual does, or does not, consider disclosure of information to be consistent with his or her reasonable expectation of privacy*.* The law ‘is not intended for the protection of the unduly sensitive’.^39^ From this perspective, reasonable expectations are to be assessed *objectively* from the perspective of the person claiming the breach but taking individual circumstances into account.

Historically, it was the perspective of the *recipient* of the information that was considered most important.^40^ However, across the past 20 years or so the importance of personal autonomy has been ‘more and more widely recognised’ by the courts.^41^ If it is self-determination that is at stake, then one might expect it to be from the patient’s perspective that a potential infringement is to be assessed. In cases since *Campbell*, the courts have indeed focused on whether in respect of the disclosed facts, the person in question had a reasonable expectation of privacy in the circumstances.^42^ Whether there was a reasonable expectation of privacy is to be judged from the perspective of a person of ordinary sensibilities placed in the position of the person to whom the information relates.

#### 2. Material Contextual Considerations

This leads us to the second consideration, what are the various features of circumstance that will be taken into account? What will inform the view of the reasonable person? On this, the courts have taken an inclusive approach. In *Murray v Express Newspapers*, Sir Anthony Clarke MR opined that,

the question whether there is a reasonable expectation of privacy is a broad one, which takes account of all the circumstances of the case. They include the attributes of the claimant, the nature of the activity in which the claimant was engaged, the place at which it was happening, the nature and purpose of the intrusion, the absence of consent and whether it was known or could be inferred, the effect on the claimant and the circumstances in which and the purposes for which the information came into the hands of the publisher.^43^

In taking this broad approach, the courts are clearly signalling an unwillingness to focus exclusively on any single contextual feature or the opinion of the individual alleging the breach. All must be taken together to determine the reasonableness of an expectation in the circumstances. There are, however, a number of things that case law indicates will be considered relevant. Here we briefly consider just three. Each is significant to later argument.

#### 3. Triviality

The courts have long indicated that law will not intervene unless the circumstances are of sufficient gravity. Historically, it was said that ‘equity ought not to be invoked merely to protect trivial tittle-tattle, however confidential’.^44^ More recently the courts have continued to recognise that ‘interference with private life had to be of some seriousness’^45^ to fall within the ambit of legal protection. The triviality of information, and its insignificance in the circumstances, will thus operate as an (internal) limit on the breadth of a duty.^46^

#### 4. Patient Information

In *Campbell v Mirror Group Newspapers Ltd*, Baroness Hale noted that information relating to an individual’s health is to be considered ‘obviously private’.^47^ However, she also said,

Not every statement about a person’s health will carry the badge of confidentiality or risk doing harm to that person’s physical or moral integrity. The privacy interest in the fact that a public figure has a cold or broken leg is unlikely to be strong enough to justify restricting the press’s freedom to report it. What harm could it possibly do?^48^

In the context of information obtained or generated within the context of a health care system, *i.e. patient* information, the courts have recognised that potential harm, even where the information itself is relatively trivial, may be to public trust in the confidentiality of the healthcare system. Baroness Hale favourably cited the European Court of Human Rights in *Z v Finland* (1997),

Respecting the confidentiality of health data is a vital principle in the legal systems of all the Contracting Parties to the Convention. It is crucial not only to respect the sense of privacy of a patient but also to preserve his or her confidence in the medical profession and in the health services in general.^49^

Thus, a healthcare context may positively contribute to the gravity of the circumstance: potentially protecting patient information where the health information in question might otherwise be considered too trivial to protect.

#### 5. Absence of Consent

In the case of *Hunter v Mann*, Boreham J considered whether a doctor owed an obligation of confidence to his or her patients and concluded:

[I]n common with other professional men … the doctor is under a duty not to disclose [voluntarily], without the consent of his patient, information which he, the doctor, has gained in his professional capacity.^50^

While this is consistent with the standard account, there have been legal developments since *Hunter* v *Mann* was reported in 1974. While the case of *Murray* did not involve patient information, it is pertinent to note that in the list of considerations set out by the court (see above) an *absence* of consent (either known or inferred) was recognised only to be one of a number of relevant considerations. Absence of consent was not suggested to be determinative.

In order to consider whether disclosure of (non-trivial) *patient* information for *individual direct care* purposes,*^51^* absent patient consent, will constitute a breach of confidence we must bring our examination of relevant case law up to date and slightly widen our lens: placing an action for breach of confidence in a broader contemporary legal landscape.

## IV. PART III—REASONABLE EXPECTATION OF PRIVACY, HUMAN RIGHTS, AND HEALTH DATA

### A. Reasonable Expectation, Privacy, and Public Benefit

Development of the protection of privacy under English law was spurred by the enactment of the Human Rights Act 1998.^52^ As a result, for at least the past 20 years, the development of the common law has been in harmony with Articles 8 and 10 of the ECHR. As far back as 2004, Lord Nicholls said in *Campbell* that the ‘time has come to recognise that the values enshrined in articles 8 and 10 are now part of the cause of action for breach of confidence’.^53^ Since then, the courts have adopted the two-part structure of Article 8 when considering actions for breach of confidence:

Article 8(1) recognises the need to respect private and family life. Article 8(2) recognises there are occasions when intrusion into private and family life may be justified. One of these is where the intrusion is necessary for the protection of the rights and freedoms of others. Article 10 (1) recognises the importance of freedom of expression.^54^

#### 1. Internal (engaged) and External (outweighed)

The approach taken by English courts is thus now parallel to the approach taken by the European Court of Human Rights (ECtHR) when considering Article 8 of the ECHR. As the Court of Appeal put it in *Murray*,

In deciding whether there is in principle an invasion of privacy, it is important to distinguish between that question, which seems to us to be the question often described as whether article 8 is engaged, and the subsequent question whether, if it is, the individual’s rights are nevertheless not infringed because of the combined effect of article 8(2) and 10 [the right to freedom of expression].^55^

In the case of *W, X, Y and Z* the same point was made by the Court using the language of ‘reasonable expectation’ and ‘public benefit’:

It is common ground that the test … involves two questions. The first is whether the claimants have a reasonable expectation of privacy in relation to the Information. This question is judged objectively by reference to the reasonable person of ordinary sensibilities. If they do have a reasonable expectation of privacy, the second question is whether there has been a breach of their rights to privacy and confidentiality. This requires a balancing exercise of weighing the public benefit that would be attained by the transmission of the Information against the harm that would result from the interference with the rights.^56^

There is then a connection between whether Article 8(1) is engaged and whether there is a reasonable expectation of privacy. Any (self-)limiting characteristic relevant to engagement of Article 8(1) will contribute to determining (internal) limits to a reasonable expectation of privacy. Whether any reasonable expectation of privacy is outweighed in the circumstances by an overriding (external) consideration will, similarly, be determined by the logic of Article 8(2).

#### 2. Personal Autonomy and Dignity: Internal Limits to Scope and Content

In *Campbell v MGN*, Lord Hoffmann noted the law’s:

new approach takes a different view of the underlying value which the law protects. Instead of the cause of action being based upon the duty of good faith applicable to confidential information and trade secrets alike, it focuses upon the protection of human autonomy and dignity – the right to control the dissemination of information about one’s private life and the right to the esteem and respect of other people.^57^

In *Re JR38*, Lord Toulson remarked,

In considering whether, in a particular set of circumstances, a person had a reasonable expectation of privacy (or legitimate expectation of protection), it is necessary to focus on both the circumstances and on the underlying value or collection of values which article 8 is designed to protect.^58^

This recognises that as the *values* enshrined in Articles 8 and 10 are now incorporated into the cause of action for breach of confidence, the values themselves will inform identification of circumstances material to whether a person has a reasonable expectation of privacy. As Lord Sumption put it in *R (Catt) v ACPO*,

Given the expanded concept of private life in the jurisprudence of the Convention, the test … must extend to every occasion on which a person has a reasonable expectation that there will be no interference with the broader right of personal autonomy recognised in the case law of the Strasbourg court.^59^

There is a parallel implication: that the law might *not* run into areas where the broader right of personal autonomy is respected and not infringed. Respect for autonomy and human dignity may operate as an internal limit to the scope and content of a reasonable expectation of privacy.^60^ In fact, this approach might account for the way the Court of Appeal dealt with the question of a reasonable expectation of privacy in relation to health data in *W, X, Y and Z*. Arguably this approach deviates from the standard account and *Hunter v Mann*: so long as human (informational) autonomy and dignity is appropriately respected, consent to disclosure may not always be required.^61^

### *B. R (on the application of W, X, Y and Z)* v *Secretary of State for Health and Home Office* [2015]

In *W, X, Y and Z*, specified limited patient information was passed by NHS Trusts to the Secretary of State for Health and then to the Home Office for the purposes of imposing immigration sanctions applicable in case of certain debts being owed to the NHS. The information contained name, gender, and date of birth of the patient and, where known, gender, current address, nationality, and travel document number with expiry date. Details about the amount and date of the debt, and the NHS trust to which it was owed, were also included.

At first instance, Mr Justice Silber considered the data to fall ‘at the least intrusive end of the spectrum of medical information’^62^ as it contained no details of illness, medical history, treatment or prognosis decided. For this reason, and due principally to the lack of clinical information contained, he decided the information did not reach the threshold of private or confidential.^63^ The Court of Appeal (CoA) disagreed with this approach. The CoA found that only if the information were, in privacy terms, ‘entirely trivial’ would it be outside the scope of protection. In the instant case it was not considered to be entirely trivial, in particular because the data in question:

reveals information of substance about the health of the data subjects, namely that they were unwell to the extent that they had to seek medical care at a particular point in time from one or more NHS bodies. […] [T]hat the data also includes charging information, which in itself reveals whether the treatment was relatively fleeting or extensive, only *reinforces* the conclusion that the Information is inherently private in nature.^64^ (emphasis added)

Thus, the Court’s approach was to consider any identifiable *patient* data, held by a doctor or a hospital medical data, to attract a reasonable expectation of privacy. Where data holds information relating to an individual’s health, even if toward the least intrusive end of the spectrum, it will ordinarily attract a duty of confidence.

The Court cited favourably professional guidance that indicates, in the medical context, sharing *any* information that is capable of identifying a patient is sharing confidential patient information:

In our view, all of these documents articulate the same approach to the issue of confidentiality: all identifiable patient data held by a doctor or a hospital must be treated as confidential. The documents have been drafted in expansive terms so as to reflect the reasonable expectations of patients that all of their data will be treated as private and confidential. These publicly available documents inform the expectations of patients being treated in the NHS. They do not seek to distinguish between more or less sensitive categories of patient data.^65^

This is consistent with the ECtHR's approach which is to recognise the right to respect to private and family life to be an expansive one.^66^ One might expect, consistent with this approach, that any disclosure of confidential information, even to parties who would themselves maintain confidentiality, would need to be authorised by a patient’s consent or justified in accordance with the provisions of Article 8(2). That is to say, one would need to identify overriding external factors (such as ‘freedom of expression’) that justified interference as ‘necessary in a democratic society’, ‘in accordance with the law’, and in pursuit of a ‘legitimate aim’. Indeed, this is the approach that the ECtHR took in *MS v Sweden*.^67^

However, crucially, this was *not* the approach taken by the Court of Appeal in *W, X, Y and Z*. Despite the fact that there was no argument that the patients had given consent to the disclosure,^68^ the Court did not consider it necessary to determine whether a breach of confidence was justifiable in the circumstances (according to external considerations and the requirements of Article 8(2)). At least, not where the patients’ reasonable expectations had been adjusted in the relevant aspect: here the finding was that Article 8 was not even engaged.^69^ Even though it was accepted that the information would ordinarily be regarded as ‘obviously private’ the Court took into account *all* the circumstances of the case, not just the absence of consent, and did not consider there to be a reasonable expectation of privacy in the relevant aspect: the disclosure to the Secretary of State for Health and the Home Office.

In particular, the Court considered significant that it was good practice for individuals to be notified of the disclosure and they were understood to have a choice as to whether to ‘accept the terms on which [treatment] was offered’.^70^ The Court reassured that the basic principle of patient confidentiality remained intact:

The present case is concerned with a particular regime under which patients are usually informed that the limited details contained in the Information [non-clinical information relating to non-resident patients] may be disclosed to a limited class of persons for a particular reason connected with immigration control. It should not be seen as a Trojan horse which will lead to the dismantling of the principle that information about a person’s health and medical treatment is inherently private and confidential.^71^

It appeared significant to the Court that the individuals could be understood to have *accepted* the disclosure: presumably consistent with respect for the values of human autonomy and dignity. The Court said:

[t]here may, however, be special circumstances where the position will be different. For example, the patient may have been admitted unconscious to the A&E Department of a hospital (for which no charges are made) and have been transferred, still unconscious, to a hospital ward (where treatment does attract charges). There may also be emergency cases where the clinical staff cannot refuse treatment and where, in practice, the patient has no choice but to accept the terms on which it is offered. Furthermore, in some cases the patient may be vulnerable and/or unable to speak English. But in most cases where the best practice [regarding notification] is followed, the patient will not have a reasonable expectation of privacy in the Information so far as the Secretary of State and the Home Office are concerned.^72^

This approach appears at variance with the standard account. There is no consent, no setting aside of the obligation of confidence by statutory authority, and no argument for an overriding public interest. This opens the door to an alternative account that holds, where the values of human autonomy and dignity are respected and *in all the circumstances* (taking into account what people are told and the opportunities they have to express a preference) there is no reasonable expectation of privacy *in the relevant aspect*, then confidential information may be disclosed without consent.

We suggest later that there may be some advantages associated with recognising this possibility. However, we raise the possibility with some hesitation for an important reason.

#### 1. Trojan Horse

Like the Court of Appeal in *W, X, Y and Z*, we do not seek to advocate an approach that will dismantle the principle that information about a person’s health and medical treatment is inherently private and confidential. It seems to us that the circumstances in which one could be confident that the use of confidential information is consistent with a respect for human autonomy and dignity are likely, minimally, to involve an individual having accessible opportunity to signal preferences in relation to uses they can freely choose to accept or reject: opt outs are available to those who wish to object. As acceptance may be more passive than consent, (in not requiring a positive signal of agreement,) appropriate demonstration of respect for autonomy and dignity might also usefully be supplemented by a requirement for additional indicators of conformity with an individual’s preferences or interests. For example, a requirement that, in addition to acceptance, the use must be consistent with previous signalled consent and dissent in other contexts (eg consent to prior stages of care) and/or the use otherwise supports an individual’s best interests and is consistent with respect for his or her autonomy and dignity (eg individual care).

For these reasons, we have some misgivings regarding the approach taken by the Court in the instant case, particularly as the use was not for individual care but for a secondary purpose. We note the relevant Guidance considered by the Court stated that:

whilst it is not necessary to seek the patient’s consent before sharing their information with the Home Office, it is best practice—if possible and appropriate—to inform them that you have done so or are going to do so and why.^73^

It does not seem clear, therefore, that each of the patients *will* have been notified, nor had accessible means to express a preference in relation to the use beyond individual care, nor that the use was freely accepted.^74^ There was no argument that it was necessary for these individuals to recognise the use to be consistent with their own interests or their own preferences. In *these* circumstances, we suggest evidence of respect for human autonomy and dignity appears thin; and so the supposition that the sharing of the confidential patient information in the circumstances did not engage Article 8(1) is poorly founded. It may have been more appropriate to consider whether there was a *public interest* in the disclosure under Article 8(2), rather than find the breadth of the duty of confidence narrowed in a way that put this aspect of private life outside the law’s protection, as we now explore.

### C. Importance of Distinguishing between Internal and External Approaches

In *Campbell*, Lord Nicholls opined that when:

Deciding what was the ambit of an individual’s ‘private life’ in particular circumstances, courts need to be on guard against using as a touchstone a test which brings into account considerations which should more properly be considered at the later stage of proportionality.^75^

The reasonableness of an expectation of privacy is *not* then to be assessed in the light of any legitimate justification for an interference with an individual’s privacy. It is, for example, not appropriate to say that there is no reasonable expectation of privacy in relation to this health data *because* intrusion is necessary to protect the rights and freedoms of others. This is to be reserved for the second stage of the test: where the duty is externally constrained. Here, any interference must satisfy the requirements of Article 8(2): interference is ‘necessary in a democratic society’, ‘in accordance with the law’, and in pursuit of a ‘legitimate aim’. These are the well-established hurdles that an individual must overcome to legitimately disclose information held under a duty of confidence.

While we should not (allow internal constraint to) provide a run-around that allows individuals to avoid appropriate application of the tests established by Article 8(2), and the Court in *W, X, Y and Z* may have opened the door wider than we would prefer, it is nevertheless important to recognise that it is possible for the touchstone test to be applied *at the first stage* (when asking if Article 8 is even engaged) and for individuals to be found to have no reasonable expectation of privacy in the circumstances *in a relevant aspect*. It is consistent with a respect for autonomy and human dignity that *W, X, Y and Z* indicates that the fact of notification and acceptance will be considered to be part of the relevant circumstance. We suggest only that the court might have applied those requirements rather more rigorously.

Where there is genuinely no reasonable expectation of privacy, due *in part* to notified disclosure being accepted by an individual, then there may be no relevant interference with personal autonomy and no reasonable expectation of privacy. Notification and acceptance are *part* of the picture.^76^ As indicators of respect for autonomy and dignity they should be accorded less weight than a real consent. Where individuals rely, not on consent to establish grounds for disclosure of confidential patient information but on ‘reasonable expectation’, then the law may, and we say should, continue to look for other positive indications of respect for autonomy and dignity (beyond notification and acceptance). In doing so, even where there is acceptance (short of consent) the law may continue to exercise a substantive restraint on action (that may be inappropriate in the face of an individual’s consent).

An important consequence of this approach is that it allows space for explicit restatement of the requirements of a consent, and reasonable expectation, in the law of confidence and consideration of the relationship with those same terms in data protection law. It enables restricted reliance upon consent (explicit or implied) in the law of confidence only where consent is real, without undermining other lawful data flows consistent with a patient’s reasonable expectation. As the requirements of valid consent in data protection law have been strengthened under new data protection law,^77^ this approach creates the space to consider the merits of aligning the requirements of a valid consent across both the law of confidence and data protection. Also, by recognising the role that reasonable expectations plays in the law of confidence, one can consider the relationship with the language of reasonable expectation under data protection law and the efficiencies that might be realised through alignment of standards, advice, and good practice across these two areas of law. Irrespective of whether these particular opportunities are seized, simply foregrounding the role that ‘reasonable expectations’ plays in the law of confidence has advantages in the substantive restraint it may place on unreasonable use and disclosure. Absent judicial consideration, we look to current guidance to establish those requirements. There is value in establishing the considerations relevant to determining the *internal* limits to a reasonable expectation with a clarity similar to the external approach and the *external* limits applied under Article 8(2). In this, current guidance falls short.

## V. PART IV—REASONABLE EXPECTATION OF PRIVACY AND CURRENT GUIDANCE

### A. Current Guidance

Current guidance (described earlier as providing the ‘standard account’), overwhelmingly assumes that data held under confidence can be disclosed only with consent, via a statutory gateway, or due to legal requirement or overriding public interest.^78^ As part I explored, this has led to consent, and implied consent in particular, doing a lot of work in the health care context.

If consent (whether explicit or implied) is to be real, then there needs to be some action or actions by the consenting individual through which consent is signalled. Asking *how* a person consented is always an appropriate question; if there is not a clear answer to how the person consented, then this may call into question whether consent is real.

It is clear that the current heavy reliance on implied consent is pushing the boundaries of claims that an individual has signalled their consent. New models of care are further widening a gap between the available evidence about the uses to which individuals have knowingly signalled consent and what in reality is happening to confidential information. As new data protection legislation^79^ beds in, and organisations pay fresh attention to the legal basis for personal data processing, there may be renewed scrutiny of the extent to which implied consent has become over-stretched in common law.^80^

Our analysis so far allows us to see that the standard account overlooks the importance of the primary consideration of whether there is a reasonable expectation of privacy in all the circumstances. Now that we have articulated the importance of reasonable expectations, we can easily make the case that the work done by the concept of implied consent in healthcare could be done by reasonable expectations. This would allow new models of care that are argued to constitute good practice, but which struggle to meet the criteria of implied consent for direct care (as expounded in Caldicott 2) to be seen to be unproblematically lawful. Moreover, as we anticipate above, by removing the pressure to classify cases under the heading of implied consent, it would allow for a conception of common law consent in healthcare that was less sharply divergent from consent under data protection legislation.^81^

Cases of sharing of confidential health information for the purposes of individual direct care within the context of a legitimate relationship that are currently taken to be justified through implied consent could equally as well be described as having reasonable expectations as their legal basis. For example, one of the grounds within the Caldicott 2 report that allow information to be shared on the basis of implied consent is that the ‘patient or client agrees to a referral from one registered and regulated health or social care professional to another’.^82^ In the circumstances of the standard case where a patient seeks the advice of a GP because of a particular ailment, and the GP refers a patient to a specialist, there is no reasonable expectation that the GP would treat relevant portions of the patient’s notes as private with respect to the specialist. As there is no reasonable expectation of privacy in all the circumstances *in the relevant aspect*; Article 8(1) is not engaged by the disclosure.

In addition to the lack of a reasonable expectation of privacy, as Caldicott 2 explains, there is also what we could describe as a *reasonable expectation of disclosure* in direct care: patients expect sharing of information to occur for individual care purposes, and ‘get frustrated if they have to answer the same questions repeatedly as they move along a care pathway’.^83^ The clear implication is that not only is it the case that such sharing does not violate a reasonable expectation of privacy, but that there are broader social norms that dictate that patients accept and expect that relevant information about their case will be shared between healthcare professionals in order to facilitate provision of care. Of course, there is nothing inherent to the test that limits application to the individual direct care context. It is possible that the conditions of ‘reasonable expectation’ could be met in relation to disclosure for purposes beyond direct care (as was held in the case in *W*, *X*, *Y and Z*). Secondary use of data has not been the focus of our contention but we note that given ‘the nature of the activity in which the claimant was engaged, the place at which it was happening, the nature and purpose of the intrusion’,^84^ the ‘reasonable expectation test’ may be more readily satisfied in case of direct care than any secondary use of data. Indeed, as we’ve already indicated—we have reservations as to whether a properly constructed test would have been met in *W*, *X*, *Y and Z*. We recognise a role for further research and professional guidance to consider this question and to explicitly articulate positive indications of ‘reasonable expectation’ that go beyond mere ‘notification and acceptance’ to ensure no interference with the underlying values of autonomy and human dignity in cases of either individual direct care or secondary use.

### B. What Difference Would Shifting from ‘implied consent’ to ‘reasonable expectations’ Make?

There are strong reasons to believe that whenever there is real consent (whether explicit or implied) to sharing of confidential patient information for a particular purpose, then *eo ipso* sharing of this information in line with what the patient has consented to does not infringe the patient’s autonomy, and does not violate a reasonable expectation of privacy. While all cases of real consent to sharing of confidential patient information are also cases where there is no violation of reasonable expectation of privacy, not all cases where there is no violation of a reasonable expectation of privacy will be cases in which consent has occurred. Because of this asymmetry, the risk that implied consent may have become overstretched, distanced from the patient or service user perspective (and that flows of confidential information may be taken to be justified in virtue of implied consent when in fact no consent has been given), provides a strong reason to shift to the lack of a reasonable expectation of privacy as a default in explaining the legal basis, at least, for the sharing of patient information for individual direct care.

In determining whether there has been a breach of confidence in cases of sharing of healthcare information, reasonable expectations have in any case played an increasing role in practice. For example, in the case of Royal Free NHS Trust and DeepMind, which we discussed in part I, the main factor that the NDG’s advice to the Information Commissioner's Office (ICO) turned on was the claim that the sharing was not in line with patients’ reasonable expectations. Thus it seems unlikely that a widespread shift from implied consent to reasonable expectations would require significant changes to good practice in informing and consulting patients. It would, however, make clearer than it has sometimes been that it is not the mere fact that an activity can be described as direct care that makes the sharing of confidential patient information lawful or not, but in the first instance whether the sharing interferes with a reasonable expectation of privacy. It brings the patient or service user’s perspective clearly to the fore. Demonstrating there to be a reasonable expectation of use, coupled with evidence of acceptance (including non-employment of accessible opportunities to object), and eg conformity with individual’s expressed preferences and interests, would establish foundations for a claim that there was no interference with a reasonable expectation of privacy in the relevant aspect. It would also provide an alternative to the proposal, contained within the published audit report of Royal Free NHS Trust and DeepMind activity, that the ‘conscience test’ is the appropriate measure of whether a duty of confidence has been breached.^85^

#### 1. The ‘Conscience Test’

We briefly turn from positive consideration of our proposed evolution of the standard account, to consider another alternative. An independent audit of activity by Royal Free NHS Trust, in relation to the project with DeepMind, considered the legality of data processing. Amongst several aspects considered was whether any duty of confidence would be breached in relation to any data flow reviewed by the Audit. As part of this, the Audit Report reviewed relevant legal tests. We strongly agree with the Report’s claim that:

the application of the law of confidence to modern health care is not straightforward. Many patients no longer have a simple bilateral relationship with a single doctor. Instead, they are treated in a complex multi-agency environment with support from different specialists using a range of information technology systems.^86^

However, there are significant flaws in the way it attempts to explicate the current law of confidence.

The Report separates consideration of the test of ‘reasonable expectations’ and the test of breach of confidence; reserving the former for questions concerning infringement of patient privacy and Article 8 ECHR.^87^ Relying upon the classic case of *Coco v AN Clark (Engineers) Ltd*^88^ to establish the circumstances under which a duty of confidence will be breached, the report suggests that when determining whether any use of confidential information is unauthorised, ‘the key question to determine whether there is a breach of confidence is: would the use trouble the conscience of a reasonable health professional’.^89^

We have no space for extensive consideration of the report, but we note authority for the ‘conscience test’ being the correct test is found in *R v Department of Health, ex parte Source Informatics,^90^* with judgment handed down in 1999. This was prior to the Human Rights Act 1998 coming into force in 2000. In the 2002 case of *A v B Plc* Lord Woolf CJ warned that ‘authorities which relate to the action for breach of confidence prior to the coming into force of the 1998 Act … are largely of historic interest only’.^91^

Indeed, the caution with which one should approach adoption of ‘the conscience test’, relied on in *Source Informatics*, as a ‘key question’ determining the breadth of a duty of confidence today, is demonstrated by the lack of reference to the case of *Source Informatics* in subsequent case law.^92^ Few of the cases cited by the Report itself reference either the case or the test.^93^ In one that does, *London Regional Transport v Mayor of London*,^94^ Sullivan J, while approving of Simon Brown LJ’s conclusions in *Source Informatics* distinguishes *Source* and the ‘conscience test’ from the content of the case.^95^ Perhaps more pertinently, Sedley LJ casts doubt on the appropriateness of continued reliance on the test. He expresses a preference for now adopting an approach that follows the structure and applies the principles (including proportionality) of Human Rights Law, rather than applying a test of conscionability:

It seems to me, with great respect, that this now well established approach [ie. the Human Rights Law approach] furnishes a more certain guide for people and their lawyers than the test of the reasonable recipient’s conscience. While the latter has the imprimatur of high authority, I can understand how difficult it is to give useful advice on the basis of it. One recipient may lose sleep a lot more readily than another over whether to make a disclosure, without either of them having to be considered unreasonable. If the test is whether the recipient ought to be losing sleep, the imaginary individual will be for practical purposes a judicial stalking-horse and the judgment more nearly an exercise of discretion and correspondingly less predictable. So for my part I find it more helpful today to postulate a recipient who, being reasonable, runs through the proportionality checklist in order to anticipate what a court is likely to decide, and who adjusts his or her conscience and conduct accordingly.^96^

There is, in fact, very little authority to support the claim that the ‘conscience test’ is to be considered the ‘key question to determine whether there is a breach of confidence’. We have already cited considerable authority for the appropriate test in this context being a ‘reasonable expectation of privacy’ and explained why the patient’s perspective should be the focus of attention rather than the professionals. However, it is not only the law that we would cite in support of our own alternative account. We see an additional advantage of the reasonable expectations standard to be the substantive restraint it places on use inconsistent with a reasonable expectation of privacy. This seems particularly valuable where, under current arrangements, parties might argue an implied consent should be assumed.

### C. Reasonable Expectations and Dissent

The fact that the reasonable expectation standard does not require consent raises important questions about the relationship between reasonable expectation and dissent. If a person explicitly dissents, then this is dispositive in cases of implied consent for individual direct care: it is not feasible to claim that a healthcare professional can continue to proceed on the basis of implied consent if the patient has explicitly withdrawn consent. It is less clear whether active dissent from a patient would always be sufficient to prevent information continuing to flow data on the basis of reasonable expectations. This is because whether there is a reasonable expectation of privacy is to be determined objectively, taking account of a wide variety of features of the case. It might seem possible that there could be an objectively reasonable expectation that data will flow, despite the fact that a patient from their subjective perspective would not agree that the expectation was reasonable. (In the case of *W, X, Y and Z*, this issue did not arise, as it was assumed that the plaintiffs had manifested acceptance towards the sharing in question.)

The difference that this gap in the treatment of dissent at the level of theory would make in practice should not be overstated, given that implied consent is sometimes currently (erroneously in our view) interpreted in guidance to allow confidential patient information to be shared on the basis of implied consent despite explicit dissent from a patient. The 2017 GMC guidance on confidentiality takes local clinical audit to have implied consent as its legal basis, but envisages that attempts will be made to dissuade patients who want to withdraw consent from local clinical audit; and that if the patient continues to object, they should be removed from the audit ‘if practicable’.^97^ Where confidential patient information may be used even in the face of a patient explicitly dissenting, then it is not clear that consent is an appropriate legal basis for the activity. The local clinical audit policy described in the GMC guidance would thus be much more naturally justified in terms of a lack of a reasonable expectation of privacy in all of the circumstances in the relevant aspect, than by the fact that patients have in fact consented to it.

We foresee that there may still be some residual worries about the idea of a shift to a reasonable expectations’ standard (related to our earlier discussion of the risk of reasonable expectations becoming a ‘Trojan horse’). It is helpful to take a little time to set out what the problem would be, if there were to be one. Our proposal is aimed to bring professional guidance and practice on confidentiality and direct care in line with legal developments, which have foregrounded not only the question of whether there is a reasonable expectation of privacy in all the circumstances, but also the range of legitimate public interests in play in deciding whether to override a reasonable expectation of privacy. To the extent that professional guidance comes apart from the law, it is usually thought to be a problem for the guidance, rather than the law. This would only underline the importance of clear leadership in this space: leadership is absent if professional guidance does not keep pace with legal developments.

In addition, we have argued that whether there is a reasonable expectation of privacy in all the circumstances needs to be interpreted in light of the animating values of ECHR Article 8—namely respect for personal autonomy and human dignity. We have argued that sharing confidential patient information autonomy without consent, yet without Article 8(1) being engaged, requires a careful attention to adequately respecting individual autonomy. Absent such careful attention Article 8(1) will be engaged, and a public interest justification will be required for overriding this reasonable expectation of privacy. Decisions to override a reasonable expectation of privacy need to take into account, inter alia, the public interest in maintaining public trust in a confidential health care system. So overall, we do not believe that a wider use of reasonable expectations in the case of individual direct care would undermine confidence in confidential health care system.^98^ Indeed, as we have noted, empirical research^99^ indicates that patients expect more sharing of data for direct care purposes than actually occurs; and have often been frustrated by lack of sharing.

## VI. CONCLUSION

We have argued that conformity with a reasonable expectation of privacy may provide an alternative legal basis to implied consent for the lawful flow of identifiable patient information. Assessment of reasonable expectations of privacy in legal cases involving patient information disclosed for individual direct care purposes is in its infancy, and so despite the fact that reasonable expectations are to be determined objectively, existing case law can give limited guidance as to the content and limits of reasonable expectations in this context. Nonetheless, the lack of case law should not be an impediment to a much wider reliance on conformity with a reasonable expectation of privacy as a legal basis for the sharing of confidential patient information, given both the need for professional guidance to take into account recent legal developments, and the increasing use of the idea of reasonable expectations within the interpretation of existing professional guidance on implied consent.

There is much to do. Guidance on reasonable expectations will need to help shape practice in two ways. First, in specifying standards to determine for example, what will indicate respect for autonomy and dignity and conformity with a reasonable expectation of privacy where there is something less than consent for disclosure, what quality of notification is sufficient? How is acceptance to be judged? How accessible do opportunities to dissent need to be? We have suggested a few of the broader considerations that may need to be taken into account where sharing of confidential patient information relies only upon acceptance (rather than consent), including consistency with an individual’s own interests and justified belief that the use is consistent with otherwise expressed preferences. These are tests more readily satisfied in relation to use for individual direct care than secondary purposes. Any extension would need to be cautious, gradual and firmly evidence based. If guidance were to reflect such an approach however, we argue, there is scope to provide better protection of autonomy and other values—most notably human dignity—than current reliance upon implied consent, particularly where such consent is not real.

Given that what often is more relevant for guidance setting in sharing of information for individual direct care is not the minimum necessary to avoid legal liability, but good practice, a second question is how the broader idea of reasonable expectations both of privacy and of disclosure could be mobilised in a way that promotes good practice in information sharing. The NDG’s call for an approach that prioritises ‘no surprises’, and which focuses on sharing identifiable information only in line with patients’ reasonable expectations of privacy and of disclosure, would be exactly what would be required by an approach that took reasonable expectations as a good practice standard. One area for future research will be to get a more in depth understanding of reasonable expectations for the use of patient information in different care contexts. The NDG recently sponsored a Citizens’ Jury to discover precisely this.^100^ Thought of in this way, the test of ‘reasonable expectation’ may be interpreted—much like the notion of ‘no surprises’ favoured by NDG—to have two dimensions. What has been done to ensure that people have reason to expect this use, and what has been done to ensure that they accept it? Such a standard will need to be partly empirically based—on what patients in fact expect and accept—but may also require some collaborative public reasoning about the kinds of expectations that do and should inform any understanding of the health service’s social contract.^101^

Above all, what is necessary is clear leadership regarding what is sufficient to establish a reasonable expectation of disclosure in circumstances that might otherwise give rise to a reasonable expectation of privacy. If we do this, then the healthcare system can move from what might become an increasingly a precarious position where a lot of information is disclosed on the basis of an ‘implied consent’—without any clear story about how individuals have in fact consented—to a securer one that licenses such sharing of confidential information on the basis of reasonable expectations.

## Footnotes

1
*R (on the application of W, X, Y and Z) v Secretary of State for Health and Secretary of State for the Home Department, the British Medical Association* [2015] EWCA Civ 1034.

2 We use the term ‘real’ to signal that a consent meets standard requirements of voluntariness, communication, permissible breadth as well as the minimum information requirements of a valid consent in the context of a duty of confidence (which may be lower than those associated with an ‘informed consent’ as understood in other contexts). See V Chico and MJ Taylor, ‘Using and Disclosing Confidential Patient Information and The English Common Law: What are the Information Requirements of a Valid Consent?’ (2018) 26(1) Med L Rev 51–72.

3 Department of Health and Social Care, ‘Confidentiality: NHS Code of Practice’ (7 November 2003) para 15 <https://www.gov.uk/government/publications/confidentiality-nhs-code-of-practice> accessed 8 October 2018 (NHS Code of Practice).

4 ibid para 16.

5 General Medical Council, ‘Confidentiality: good practice in handling patient information’ (January 2017), para 9 <https://www.gmc-uk.org/ethical-guidance/ethical-guidance-for-doctors/confidentiality> accessed 8 October 2018 (Confidentiality: good practice in handling patient information).

6 ibid para 10.

7 NHS Code of Practice (n 3), ‘Glossary’. The precise requirements for an explicit consent are subject to disagreement. As our focus is on cases that uncontroversially do not involve explicit consent, we do not engage with these debates here. While our focus is the common law, it is worth noting that—while consent is *not* likely to be the legal basis in data protection law for the processing of data for healthcare purposes (see Information Commissioner’s Office, ‘General Data Protection Regulation (GDPR) FAQs for small health sector bodies’ <https://ico.org.uk/for-organisations/health/health-gdpr-faqs/> accessed 8 October 2018 (General Data Protection Regulation FAQs))—there is a trend towards tightening the requirements of an explicit consent more generally. The General Data Protection Regulation requires that it be ‘a clear affirmative act establishing a freely given, specific, informed and unambiguous indication of the data subject's agreement to the processing of personal data relating to him or her’: General Data Protection Regulation, Recital 32.

8 According to the GMC guidance, ‘Implied consent refers to circumstances in which it would be reasonable to infer that the patient agrees to the use of the information, even though this has not been directly expressed.’: Confidentiality: good practice in handling patient information (n 5) para 13. See also *O’Brien* v *Cunard SS Co Ltd* 57 Mo L Rev (1992)

9 It is controversial whether hypothetical consent provides either a moral or a legal justification. To the extent that it does provide such a justification, this is in spite the fact that no relevant actual consent has occurred. For further discussion, see David Enoch, ‘Hypothetical Consent and the Value (s) of Autonomy’ (2017) 128(1) Ethics 6.

10 Note that the Information Commissioner’s Office (ICO) in the UK has indicated that implied consent, as relied upon in the NHS, would not satisfy the requirements of a valid consent for the purposes of data protection law: ‘This type of assumed implied consent would not meet the standard of a clear affirmative act – or qualify as explicit consent for special category data, which includes health data’. Information Commissioner’s Office ‘Lawful basis for processing: Consent’ <https://ico.org.uk/for-organisations/guide-to-the-general-data-protection-regulation-gdpr/consent/> accessed 13 October 2018, 10. The significance of this is undercut by the fact that consent is not likely to be the lawful basis for processing under data protection law in any case. See General Data Protection Regulation FAQs (n 7).

11 Chico and Taylor (n 2) 51–72.

12 ibid.

13
NHS Code of Practice (n 3) 11.

14 The public attitudes work done by IPSOS Mori on behalf of the GMC to support the 2017 Confidentiality guidelines suggests this. Amongst the questions asked was whether the patient would expect doctors to rely on implied consent to reveal confidential information to administrative staff at the hospital or GP practice who were supporting patient care, by typing discharge letters. Responses from publics consulted were mixed, ‘with some participants stating that they would expect (and be comfortable with) doctors relying on implied consent. Others were comfortable with some information being shared with these (for example name and address information) but not medical information, without direct consent, whilst others said ‘No’ to implied consent altogether. Reasons given were lack of trust, fear of being judged and a higher risk of information being leaked’. See Ipsos MORI, ‘Exploring patient and public attitudes towards medical confidentiality: Findings from Discussion Groups and In-depth Interviews 2016’ (Ipsos MORI, April 2016) 7 <https://www.gmc-uk.org/-/media/documents/Exploring_patient_and_public_attitudes_towards_medical_confidentiality_FINAL_270416.pdf_65939141.pdf> accessed on 8 October 2018.

15 See eg the 2014 Memorandum of Understanding between NHS Commissioning Board (NHS England) and the General Pharmaceutical Council where both organisations committed to the principle ‘collect once, use many’, ‘as a means to reducing the burden of regulation’ 2 <https://www.pharmacyregulation.org/sites/default/files/nhse_gphc_mou_290414.pdf> accessed 9 October 2018.

16 Caldicott Review: Information governance in the health and care system (NHS, 26 April 2013), 36 (Information Governance Review).

17 The Review defined direct care as ‘A clinical, social or public health activity concerned with the prevention, investigation and treatment of illness and the alleviation of suffering of individuals … undertaken by one or more registered and regulated health or social care professionals and their team with whom the individual has a legitimate relationship for their care’ 128.

18 See for example, BMA 2013, *Principles for sharing and accessing local shared electronic patient records for direct patient care*, <https://www.bma.org.uk/advice/employment/ethics/confidentiality-and-health-records/principles-for-sharing-electronic-patient-records> accessed 7 April 2019. GMC, *Confidentiality: good practice in handling patient information* (2017) para 28 makes a very similar distinction, though without invoking the idea of ‘legitimate relationship’.

19 Information Governance Review (n 16) 36.

20 See the Data Protection Act 1998 Undertaking signed by the Royal Free at the close of the ICO investigation, paras 10 and 15 <https://ico.org.uk/media/action-weve-taken/undertakings/2014352/royal-free-undertaking-03072017.pdf> accessed on 15 October 2018.

21 See J Powles and H Hodson, ‘Google DeepMind and Healthcare in an Age of Algorithms’ (2017) 7(4) Health & Technol, 351, 351–67; J Powles and H Hodson, ‘Response to DeepMind’ (2018) 8 Health & Technol 15, 15–29.

22 NDG letter to Royal Free, December 2016 <https://assets.publishing.service.gov.uk/government/uploads/system/uploads/attachment_data/file/633298/6._Ref_534_NDG_Letter_to_Royal_Free_FINAL_December_2016.pdf> accessed 15 October 2018.

23 Legitimate relationship is defined on page 39 of the 2013 Caldicott 2 report. See also, National Data Guardian (2017), ‘Developing a consensus on data sharing to support NHS clinical genetics and genomics services’, <https://assets.publishing.service.gov.uk/government/uploads/system/uploads/attachment_data/file/644689/546_Developing_a_consensus_on_data_sharing_to_support_NHS_clinical_genetics_and_genomics_services_FINAL.pdf> accessed 15 October 2018.

24 For the idea of a ‘social licence’ see P Carter, G Laurie and M Dixon-Woods, ‘The Social Licence for Research: Why *care.data* Ran Into Trouble’ (2015) 41 J of Med Ethics 404–09.

25
*Campbell v Mirror Group Newspapers Ltd* [2004] UKHL 22, [2004] All ER 995, [13–14]. Although Lord Nicholls (and Lord Hoffmann) were in the minority in *Campbell*, the difference of opinion was acknowledged to be on the facts. Lord Hoffmann stated that he could not discern any significant differences between the views of the members of the appellate committee regarding the applicable principles. See: *Campbell v Mirror Group Newspapers Ltd* [36] (Lord Hoffmann) (n 25).

26
*Murray v Express Newspapers plc*, [2008] EWCA Civ 446, [24]. See also *Campbell* v *Mirror Group Newspapers Ltd* (n 25); *R (Wood)* v *Commissioner of Police of the Metropolis* [2009] EWCA Civ 414, [2010] 1 WLR 123.

27
*Re JR38’s Application for Judicial Review* [2015] UKSC 42, [2016] AC 1131, [55] (Lord Clarke). He was concerned with it occupying the position of a necessary precondition for protection [61]. See also esp. [55–60].

28 ibid [54].

29 We address an alternative account, that would see the ‘conscience test’ as the ‘key test’, under Part IV.

30
*Re JR38* [106] (Lord Clarke) (n 27).

31 ibid [61].

32 See eg *Coco v AN Clark (Engineers) Limited* [1969] RPC 41, [1968] FSR 415; *Campbell* v *Mirror Group Newspapers Ltd* (n 25); *Murray v Express Newspapers plc* [2008] EWCA Civ 446, EMLR 12; *Re JR38* (n 27).

33 In *W v Egdell* [1989] Ch 359, [1990] 2 WLR 471 sharing a medical report with ‘the responsible authorities’ was viewed differently to selling it to a newspaper, p 419 (Bingham LJ). See also *Initial Services v Putterill* [1968] 1 QB 396, [1967] 3 All ER 145, a149.

34 The distinction anticipates the structure of argument under art 8, introduced in part III of this article. In practice it is not always easy to place specific considerations in a category. For example, the ‘inequity rule’ articulated in *Gartside v Outram* [1857] LJ Ch 113, started as an internal principle limiting the breadth of a duty owed, but there has been a move (traceable at least to *Initial Services v Putterill*, ibid) toward assessing the public interest defence in disclosure as an overriding consideration (See K Koomen, ‘Breach of Confidence and the Public Interest Defence: Is it in the Public Interest?’ (1994) 10 QUT L Rev 56). As we will see though, the distinction is now crucial to an understanding of the tests to be applied to limit privacy protection under human rights law.

35 Although, in the case of non-triviality, there is a suggestion that one might assess whether an intrusion meets ‘a certain level of seriousness’ independent of whether there is a reasonable expectation of privacy (see *R (Wood) v Commissioner of Police of the Metropolis* (n 26), [22] (Laws LJ)) nothing turns, for the sake of our analysis, on whether triviality is separated assessed or folded into the broad question posed in *Murray*. In either case one may demonstrate *internal* limits to the scope of any duty owed.

36
*Campbell v Mirror Group Newspapers Ltd* (n 25), [14].

37 ibid [99] (Lord Hope).

38 ibid [99] (Lord Hope of Craighead).

39 ibid [94] (Lord Hope).

40 In the classic case of *Coco v AN Clark (Engineers) Limited* (n 32), 421, Megarry J opines that ‘it may be that the hard-worked creature, the reasonable man, may be pressed into service once more for I do not see why he should not labour in equity as well as at law. It seems to me that if the circumstances are such that any reasonable man standing in the shoes of the recipient of the information would have realised that upon reasonable grounds the information was given to him in confidence, then this should suffice to impose upon him the equitable obligation of confidence’. See also comments in *R* v *Department of Health, ex parte Source Informatics (No 1)* [1999] EWCA Civ 3011, [2001] FSR 8, [31] (Simon Brown LJ) discussed further in part IV of this article.

41
*Montgomery* v *Lanarkshire Health Board (Scotland)* [2015] UKSC 11, WLR 768, [68]. See also *Campbell* v *Mirror Group Newspapers Ltd* (n 25) esp [50]–[51], [134]; *R (Wood) v Commissioner of Police of the Metropolis* (n 26), [20–23]; R (*Catt) v Association of Chief Police Officers of England, Wales and Northern Ireland (Equality and Human Rights Commission Intervening)* [2015] UKSC 9, WLR 664, [4].

42 See eg *Murray v Express Newspapers plc* (n 32), esp [24], [31], [35]; *R (Wood) v Commissioner of Police of the Metropolis* (n 26); R (*Catt) v Association of Chief Police Officers of England*, ibid; *Re JR38* (n 27).

43
*Murray v Express Newspapers plc* (n 32) [36].

44
*Coco* v *A N Clark (Engineers) Limited* (n 32) 421.

45
*McKennit v Ash* [2006] EWCA Civ 1714, [2008] QB 73, [12].

46 Whether that operates as a limiting feature independent of the test of reasonable expectations (*R (Wood) v Commissioner of Police of the Metropolis* (n 26) [22], [23] (Laws LJ)) or as part of it (*R (on the application of W, X, Y and Z)* (n 1) [34–35]) does not affect our argument.

47
*Campbell v Mirror Group Newspapers Ltd* (n 25) [136].

48 ibid [157].

49 ibid [145]. See also *Z v Finland* (1998) 25 EHRR 371, [1997] ECHR 10, [95]; *MS* v *Sweden* 20837/92, [1999] 28 EHRR 313, 3 BHRC 248, [41].

50
*Hunter v Mann* [1974] QB 767, [1974] 2 WLR 742, 772.

51 Note that, even in *Hunter v Mann* the disclosure in question was not to another healthcare professional for the purposes of an individual’s care. The disclosure was to the police in relation to investigation of a road traffic accident.

52
*Campbell v Mirror Group Newspapers Ltd* (n 25) [11] (Lord Nicholls).

53 ibid [17] (Lord Nicholls).

54 ibid [20] (Lord Nicholls).

55
*Murray v Express Newspapers plc* (n 32) [24].

56
*R (on the application of W, X, Y and Z)* (n 1) [26].

57
*Campbell v Mirror Group Newspapers Ltd* (n 25) [51]. See also *Douglas* v *Hello* [2001] QB 967, [126], [2001] 2 WLR 992 (Sedley LJ).

58
*Re JR38* (n 27) [97] (Lord Toulson).

59
*R (Catt) v Association of Chief Police Officers of England, Wales and Northern Ireland (Equality and Human Rights Commission Intervening)* (n 41) [91].

60 This is consistent with Lord Hope's (Deputy President of the Supreme Court) comment that where ‘a person knowingly or intentionally involves himself in activities which may be recorded or reported in public, in circumstances where he does not have a reasonable expectation of privacy’ art 8 is not engaged (*Kinloch v HM Advocate (Scotland)* [2012] UKSC 62, [19], [2013] 2 WLR 141).

61 Extensive consideration of the relationship between autonomy, human dignity and privacy must sit outside the scope of this article but we briefly reflect here that there is a conception of (informational) privacy that would insist on particular ‘norms of exclusivity’ being respected (See M Taylor, *Genetic Data and the Law* (CUP 2012) 14–26). Interference with such norms would be a failure to respect a reasonable expectation of a private life. While norms must expect some kind of control to be exerted over information flows, that control need not necessarily be exerted by the subject of the intrusion. (A peeping tom may infringe privacy even when the subject of their gaze is sleeping and has no prospect of control.) In recognising a respect for autonomy *and* human dignity as values protected by art 8, the courts are recognising the law to be capable of upholding *both* normative expectations of individual self-determination and respect for norms of exclusivity where an individual has no prospect of control (but a failure to respect the norm would be a failure to respect human dignity).

62
*R (on the application of W, X, Y and Z) v Secretary of State for Health and Secretary of State for the Home Department* [2014] EWHC 1532 (Admin) [45].

63 ibid [46], [47].

64
*R (on the application of W, X, Y and Z)* (n 1) [35].

65 ibid [39].

66
*Von Hannover v Germany* [2004] EMLR 21, (2005) 40 EHRR 1.

67 An individual’s health records had been supplied by a clinic to the Swedish Social Insurance Office, without her consultation, to investigate a claim made for statutory compensation. The disclosure *was* considered to be an interference with the applicant’s right to respect for private life guaranteed by art 8(1). The Court then went on to consider whether the interference was justified under 8(2). *MS v Sweden* (1999) 28 EHRR 313.

68 The Guidance supporting delivery of the Charging Regulations was amended in 2014 to read ‘whilst it is not necessary to seek the patient’s consent before sharing their information with the Home Office, it is best practice – if possible and appropriate – to inform them that you have done so or are going to do so and why’ *R (on the application of W, X, Y and Z) v The Secretary of State for Health v The Secretary of State for the Home Department* (n 1), [20].

69 The Court did support a balancing exercise that would find, consistent with the requirements of art 8(2), in favour of disclosure in the circumstances in cases where art 8(1) *was* engaged. See *R (on the application of W, X, Y and Z)* (n 1) [48–51].

70
*R (on the application of W, X, Y and Z)* (n 1) [45].

71 ibid [46].

72 ibid [45].

73 ibid [20] (Briggs LJ).

74 ibid [47].

75
*Campbell v Mirror Group Newspapers Ltd* (n 25) [21] (Lord Nicholls).

76 There may be other considerations beyond the scope of this article. See eg comments in *Re JR38* (n 27) [110] (Lord Clarke).

77 Data Protection Act 2018 and General Data Protection Regulation (EU) 2016/679 OJ L119 (GDPR).

78 See eg Confidentiality: good practice in handling patient information (n 5) paras 80–112.

79 n 77.

80 Although consent may not be the lawful basis for processing under art 6 or art 9 GDPR, in order for processing to be lawful under data protection legislation, it is necessary for other legal requirements to be met. This will include, for example, compliance with the law of confidence. See: Information Commissioner’s Office, ‘Principle (a): Lawfulness, fairness and transparency’ (ICO) <https://ico.org.uk/for-organisations/guide-to-the-general-data-protection-regulation-gdpr/principles/lawfulness-fairness-and-transparency/> accessed 7 April 2019.

81 Information Commissioner’s Office (n 7).

82 Information Governance Review (n 16) 39.

83 ibid 35. Healthwatch Surrey’s report ‘If I’ve told you once … . People’s views on record sharing between the health and care professionals involved in their care in Surrey’ (Healthwatch Surrey, October 2015) found further evidence of frustration from having to repeat the same story to different health care professionals. See <https://www.healthwatchsurrey.co.uk/wp-content/uploads/2017/06/If-Ive-told-you-once-Peoples-views-on-record-sharing-between-health-and-social-care-professionals-FULL-REPORT.pdf> accessed 15 October 2018.

84 n 55.

85 See: Linklaters LLP, ‘Audit of the acute kidney injury detection system known as Streams: The Royal Free London NHS Foundation Trust’ (Linklaters, 7 June 2018) <http://s3-eu-west-1.amazonaws.com/files.royalfree.nhs.uk/Reporting/Streams_Report.pdf> accessed 15 October 2018.

86 Linklaters LLP, *Audit of the acute kidney injury detection system known as Streams* (17 May 2018) [30].

87 This is despite, in *Murray v Express Newspapers plc* (n 32) [27] Sir Anthony Clarke MR favourably citing Buxton LJ, [in the case of *McKennitt v Ash* (n 45)] with whom Latham and Longmore LJJ agreed, [making] the point that arts 8 and 10 of the Convention are now the very content of the domestic tort that the English court must enforce.

88
*Coco v AN Clark (Engineers) Limited* (n 32).

89 Information Governance Review (n 16) para 31.4.

90
*R v Department of Health, ex parte Source Informatics Ltd (No 1)* [1999] EWCA Civ 3011, [2000] 2 WLR 940.

91
*A v B Plc* [2002] EWCA Civ 337, [9], [2002] 3 WLR 542.

92 None of these Supreme Court cases, decided subsequent to the Human Rights Act 1998 coming into force, relies upon the ‘conscience test’ described in the report or reference *Source Informatics* (n 88) in the judgments. Each invokes the test of ‘reasonable expectation of privacy’: *Campbell v Mirror Group Newspapers Ltd* (n 25); *Kinloch v HM Advocate* (*Scotland*) (n 60); *R (Catt) v Association of Chief Police Officers* (n 41); *Re JR38* (n 27).

93 In a cursory examination of each of the cases cited by the relevant sections of the report (31.1 to 31.8) we could only find reference to *Source Informatics* (n 90) in *London Regional Transport v Mayor of London* [2001] EWCA Civ 1491, [2003] EMLR 4.

94 ibid.

95 ibid [47].

96 ibid [58].

97 Confidentiality: good practice in handling patient information (n 5) para 97.

98 There may also be grounds for going beyond the protection of confidential patient information that would be required by the law—as a matter of policy adopting the rule that if a patient objects to information being shared for their direct care, then this dissent should be sufficient to prevent sharing of their information, even if hypothetically doing so would have been allowed under the equitable action for breach of confidence. For analogous reasons, the Confidentiality Advisory Group (CAG), which makes recommendations to the Secretary of State for Health to set aside the duty of confidence for medical purposes that are beyond direct care and are in the public interest, has taken the position that, because of the important public interest of maintaining trust in a confidential health system, it is a standard condition of support that individual dissent is respected. See NHS Health Research Authority, ‘Standard Conditions of Support’ (17 August 2018) <https://www.hra.nhs.uk/about-us/committees-and-services/confidentiality-advisory-group/guidance-confidentiality-advisory-group-applicants/standard-conditions-support/> accessed 16 October 2018. See also MJ Taylor, ‘Legal Bases for Disclosing Confidential Patient Information for Public Health: Distinguishing between Health Protection and Health Improvement’ (2015) 23(3) Med L Rev 348–74.

99 See P Singleton and others, *General Medical Council: Public and Privacy Attitudes to Privacy of Healthcare Data – A Survey of the Literature* (Cambridge Health Analytics 2007) 17.

100 Office for the National Data Guardian and Connected Health Cities, ‘Reasonable Expectations Report: A report of a citizens’ jury designed to explore when it is reasonable for patients to expect patient data to be shared’ (April 2018) <https://www.connectedhealthcities.org/wp-content/uploads/2018/08/Reasonable-expectations-jury-report-v1.0-FINAL-09.08.18.pdf> accessed 13 October 2018.

101 See A Lucassen, J Montgomery and M Parker, ‘Ethics and the Social Contract for Genomics in the NHS’, *Generation Genome: Annual report of the Chief Medical Officer 2016* (2016) ch 16 <https://assets.publishing.service.gov.uk/government/uploads/system/uploads/attachment_data/file/631043/CMO_annual_report_generation_genome.pdf> accessed 16 October 2018.

## Notes

The authors would like to acknowledge the extremely useful comments made on draft versions of this piece by: Joanne Bailey, John Carvel, Victoria Chico, Alan Hassey, Graeme Laurie, Miranda Mourby, Eileen Phillips, Megan Richardson, Harriet Teare, David Townend and the two anonymous reviewers. James Wilson’s research was supported by the i-‐sense EPSRC IRC in Early Warning Sensing Systems for Infectious Diseases (Grant reference number EP/K031953/1, <http://www.i-sense.org.uk>).

