# Reasonable Expectations of Privacy and Disclosure of Health Data

**DOI:** 10.1093/medlaw/fwz021

**Published:** 2019-08-14

**Authors:** Mark J Taylor, James Wilson

**Affiliations:** 1 Associate Professor in Health Law and Regulation, Melbourne Law School, University of Melbourne, Australia; 2 Senior Lecturer, Department of Philosophy, UCL, London, UK

*Medical Law Review*, 2019, doi:10.1093/medlaw/fwz009

In the original version of this article, the following funding acknowledgement was mistakenly omitted:

James Wilson's research was supported by the i‐sense EPSRC IRC in Early Warning Sensing Systems for Infectious Diseases (Grant reference number EP/K031953/1, <http://www.i-sense.org.uk>).

The above acknowledgement has now been added to the paper; the authors would like to apologise for its initial omission.

